# Evolution of naturally occurring 5'non-coding region variants of Hepatitis C virus in human populations of the South American region

**DOI:** 10.1186/1743-422X-4-79

**Published:** 2007-08-02

**Authors:** Gonzalo Moratorio, Mariela Martínez, María F Gutiérrez, Katiuska González, Rodney Colina, Fernando López-Tort, Lilia López, Ricardo Recarey, Alejandro G Schijman, María P Moreno, Laura García-Aguirre, Aura R Manascero, Juan Cristina

**Affiliations:** 1 Laboratorio de Virología Molecular. Centro de Investigaciones Nucleares. Facultad de Ciencias, Iguá 4225, 11400 Montevideo, Uruguay; 2 Laboratorio de Virología, Departamento de Microbiología, Facultad de Ciencias, Pontificia Universidad Javeriana, Cra 7 # 43-82 Ed 50 of 313, Bogotá, Colombia; 3 Facultad de Ciencias Médicas y Bioquímicas, Universidad Mayor de San Andrés, Av. Villazón No. 1995 Monoblock Central, La Paz, Bolivia; 4 Laboratorio de Biología Molecular, Grupo CentraLab, Buenos Aires, Argentina; 5 Instituto de Investigaciones en Ingeniería Genética y Biología Molecular, Vuelta de Obligado 2490, Second Floor, 1428 Buenos Aires, Argentina; 6 Department of Biochemistry and McGill Cancer Center, McGill University, Montreal, Quebec, Canada H3G 1Y6

## Abstract

**Background:**

Hepatitis C virus (HCV) has been the subject of intense research and clinical investigation as its major role in human disease has emerged. Previous and recent studies have suggested a diversification of type 1 HCV in the South American region. The degree of genetic variation among HCV strains circulating in Bolivia and Colombia is currently unknown. In order to get insight into these matters, we performed a phylogenetic analysis of HCV 5' non-coding region (5'NCR) sequences from strains isolated in Bolivia, Colombia and Uruguay, as well as available comparable sequences of HCV strains isolated in South America.

**Methods:**

Phylogenetic tree analysis was performed using the neighbor-joining method under a matrix of genetic distances established under the Kimura-two parameter model. Signature pattern analysis, which identifies particular sites in nucleic acid alignments of variable sequences that are distinctly representative relative to a background set, was performed using the method of Korber & Myers, as implemented in the VESPA program. Prediction of RNA secondary structures was done by the method of Zuker & Turner, as implemented in the *mfold *program.

**Results:**

Phylogenetic tree analysis of HCV strains isolated in the South American region revealed the presence of a distinct genetic lineage inside genotype 1. Signature pattern analysis revealed that the presence of this lineage is consistent with the presence of a sequence signature in the 5'NCR of HCV strains isolated in South America. Comparisons of these results with the ones found for Europe or North America revealed that this sequence signature is characteristic of the South American region.

**Conclusion:**

Phylogentic analysis revealed the presence of a sequence signature in the 5'NCR of type 1 HCV strains isolated in South America. This signature is frequent enough in type 1 HCV populations circulating South America to be detected in a phylogenetic tree analysis as a distinct type 1 sub-population. The coexistence of distinct type 1 HCV subpopulations is consistent with quasispecies dynamics, and suggests that multiple coexisting subpopulations may allow the virus to adapt to its human host populations.

## Background

Hepatitis C virus (HCV) has infected an estimated 170 million people worldwide and therefore creates a huge disease burden due to chronic, progressive liver disease [1]. Infections with HCV have become a major cause of liver cancer and one of the most common indications for liver transplantation [2-4]. The virus has been classified in the family *Flaviviridae*, although it differs from other members of the family in many details of its genome organization [2].

HCV is an enveloped virus with an RNA genome of approximately 9400 bp in length. Most of the genome forms a single open reading frame (ORF) that encodes three structural (core, E1, E2) and seven non-structural (p7, NS2-NS5B) proteins. Short untranslated regions at each end of the genome (5'NCR and 3'NCR) are required for replication of the genome. This process also requires a *cis*-acting replication element in the coding sequence of NS5B recently described [5]. Translation of the single ORF is dependent on an internal ribosomal entry site (IRES) in the 5'NCR, which interacts directly with the 40S ribosomal subunit during translation initiation [6].

Comparison of nucleotide sequences of variants recovered from different individuals and geographical regions has revealed the existence of six major genetic groups [1]. Each of the six major genetic groups of HCV contains a series of more closely related sub-types.

Little is known about the earlier divergence of the six major genotypes of HCV, the origins of infection in humans and the underlying bases of the current geographical distribution of genotypes. Some genotypes, such as 1a, 1b or 3a have become widely distributed and now are responsible for the vast majority of infections in Western countries [2].

Genotype 1 is the most prevalent type in the Latin American region [7]. Previous and recent studies on genetic variation of HCV revealed a diversification of type 1 HCV strains circulating in that region [8-12]. There is no knowledge about the degree of genetic variability of HCV strains circulating in Bolivia and Colombia. This study aimed to elucidate these matters by performing a phylogenetic analysis of 5'NCR sequences from type 1 HCV strains recently isolated in Bolivia, Colombia and Uruguay, as well as available comparable sequences of HCV strains isolated in other regions of South America. In order to compare the results found for the South American region with other regions of the world, the same approach was used to perform a phylogenetic analysis of HCV strains isolated in Europe and North America.

## Results

### Phylogenetic tree analysis of HCV strains isolated in the South American region

**Figure 1:**
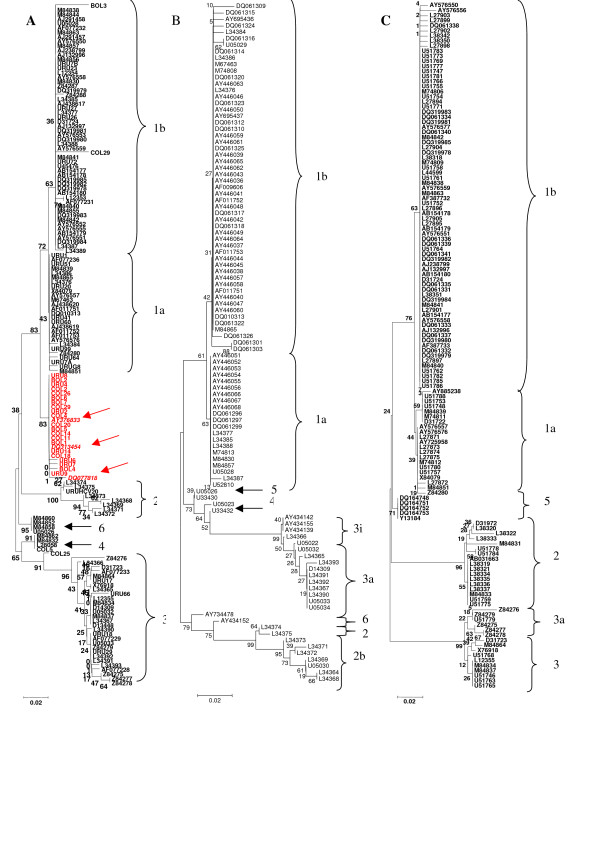
**Phylogenetic analysis of 5'NCR sequences of HCV strains**. Strains in the trees are shown by their accession numbers for strains previously described and their genotypes are indicated at the right side of the figure. Bolivian, Colombian and Uruguayan strains are shown by name. Number at the branches show bootstrap values obtained after 1000 replications of bootstrap sampling. Bar at the bottom of the trees denotes distance. In (A) the phylogenetic tree for HCV strains isolated in South America is shown. Strains assigned to a newly genetic lineage in HCV type 1 cluster are shown in red. Argentinean strains [EMBL:DQ077818] (Schijman *et al*., unpublished data), [EMBL:DQ313454] and [EMBL:AY376833] (Gismondi et al. [8, 9] previously reported as a new genetic lineage inside type 1 strains are shown in italics and an arrows denote its position in the figure. Phylogeny for HCV strains isolated in North America and Europe are shown in (B), (C), respectively.

To study the degree of genetic variation of HCV strains isolated in Bolivia and Colombia, sequences from the 5'NCR of Bolivian, Colombian and Uruguayan strains recently isolated by us, as well as all available comparable sequences (i.e. longer than 220 nucleotides) from HCV strains isolated in the South American region were aligned. Once aligned, phylogenetic trees were created by the neighbor-joining method applied to a distance matrix obtained under the Kimura two-parameter model [13]. As a measure of the robustness of each node, we employed the bootstrap method (1000 pseudo-replicas). The results of these studies are shown in Fig. 1A.

All HCV strains included in this study are clustered according to their genotype. Inside the main cluster of type 1 strains, different genetic lineages can be observed. One main line represents sub-type 1b strains (Fig. 1A, upper part), another represents type 1a strains (Fig. 1A, middle). Interestingly, type 1 HCV strains isolated in Bolivia, Colombia and some of the Uruguayan strains do not clustered together with major type 1 sub-types (1a and 1b). Instead, they are assigned to a different genetic lineage together with strains [EMBL:DQ077818], [EMBL:AY376833] and [EMBL:DQ313454], recently reported by Gismondi et al.[8,9] and Schijman et al. (EMBL database submissions) as a new type 1 genetic lineage circulating in Argentina (see Fig. 1A, middle, cluster in red).

To observe if similar results can be found in other geographic regions of the world, the same studies were carried out for strains isolated in North America and Europe. The results of these studies are shown in Figs. 1B and 1C, respectively.

As it can be seen in the figures, while three different clusters can be clearly identified in HCV type 1 strains isolated in South America, this is not observed for type 1 strains isolated in North America or Europe (compare Fig. 1A with Figs. 1B and 1C).

### Signature pattern analysis of type 1 HCV strains isolated in South America

**Figure 2:**
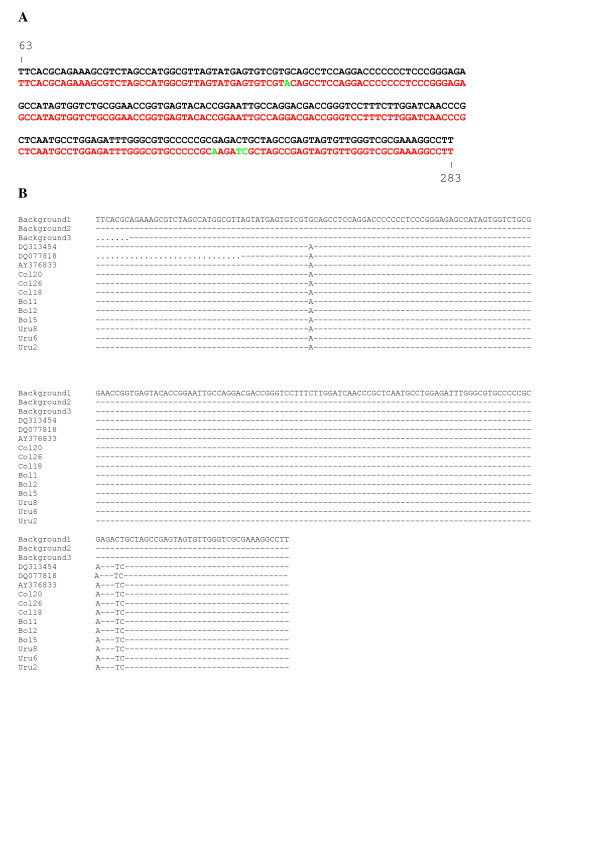
**Signature pattern analysis of type 1 HCV strains isolated in South America**. In (A) the consensus nucleotide sequence in the background set of type 1 HCV strains isolated in South America is shown in black. The consensus nucleotide sequence in the query (signature sequence) set is shown in red. Query sequence signature identified by VESPA is shown in green. Numbers in the figure shows IRES nucleotide positions, relative to strain HCV1b [16]. In (B) an alignment of 5'NCR sequences from strains belonging to the third cluster observed in type 1 HCV strains isolated in the South American region with corresponding consensus sequences of type 1 HCV strains isolated in South America (Background1), Europe (Background 2) or North America (Background3) is shown. Strains are shown by accession number for strains previously described, or by name at the left side of the figure. Identity to consensus sequences is indicated by a dash. Gaps introduced during alignment are indicated by a dot.

In order to test if the presence of the third phylogenetic lineage in type 1 HCV strains isolated in South America was due to a particular sequence signature, present exclusively in HCV strains assigned to that lineage, a signature pattern analysis was performed to assess viral sequence relatedness. For that purpose, a query dataset of 19 type 1 HCV sequences belonging to this third cluster was analyzed using a background dataset of 19 type 1 HCV sequences assigned to the two other clusters found in the South American region (see Fig. 1A). The results of these studies detected the presence of a sequence signature in type 1 HCV strains assigned to the third genetic lineage in the phylogenetic tree analysis (Fig. 2). Comparison of the frequencies obtained for each particular nucleotide and position in the signature gives statistical support to these findings (Table 1). When similar studies were performed using the same query dataset and background datasets of sequences from strains isolated in Europe or North America, similar results were obtained (Table 1). These results suggest that the sequence signature found in HCV type 1 strains isolated in South America may be characteristic of this geographic region of the world. To observe if this nucleotide sequence signature can be found indeed in strains isolated outside the South American region, BLAST studies were performed using sequences from strains bearing the sequence signature as a query against all HCV strains reported to HCV LANL Database [14]. Only strains isolated in the South American region have 100% similarity to the signature sequence strains (not shown).

**Table 1:** Frequencies of signature nucleotides identified in the 5'NCR of type 1 HCV strains isolated in South America^a^

	Frequency of query nucleotides				Frequency of background nucleotides			
	
Position^b^:	107	243	247	248	107	243	247	248
Nucleotide:	A	A	T	C	G	G	C	T
	
Among query set:	0.947	0.947	0.947	0.947	0.053	0.053	0.053	0.053
Among background set 1:	0.000	0.421	0.000	0.000	1.000	0.579	1.000	1.000
Among background set 2:	0.000	0.316	0.105	0.105	1.000	0.684	0.895	0.895
Among background set 3:	0.158	0.368	0.000	0.000	0.842	0.632	1.000	1.000

^a ^Background sets 1, 2 and 3 are composed by type 1 HCV strains isolated in South America, Europe and North America, respectively.

^b ^Numbers refer to nucleotide sequence position relative to strain HCV1b sequence [14].

### Prediction of secondary structure of signature RNA sequences

**Figure 3:**
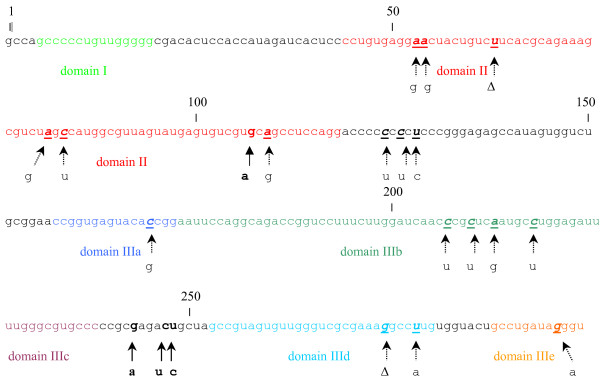
**HCV IRES mutations found in sequence signature strains isolated in South America**. The 5'NCR sequences of strain HCV1b [16] is shown. The locations of the nucleotide mutations found in the sequence signature are shown in bold and a solid arrow indicates each particular substitution. Sequences previously identified to belong to a specific IRES domain [16] are indicated by colours and domain number is indicated bellow the sequence. IRES nucleotide substitutions positions previously reported in the literature [16] or in the HCV Database [14] are indicated in bold italics underlined. Each particular previously reported substitution is indicated by a dotted arrow. Δ means deletion. Numbers in the figure denote nucleotide position in HCV sequence according to strain HCV1b [16].

Biochemical and functional studies have revealed that the 5'NCR of HCV folds into a highly ordered complex structure with multiple stem-loops [15]. This complex RNA structure contains four distinct domains, with domains II, III and part of domain IV forming the IRES. These highly folded secondary RNA elements function as *cis-*signals for interaction with the 40S ribosome subunit and/or eukaryotic translation initiation factors [6]. Signature mutations map in IRES stem-loops II (G107A) and III (G243A, C247U and U248C) relative to strain HCV1b [16] (see Fig. 3).

**Figure 4:**
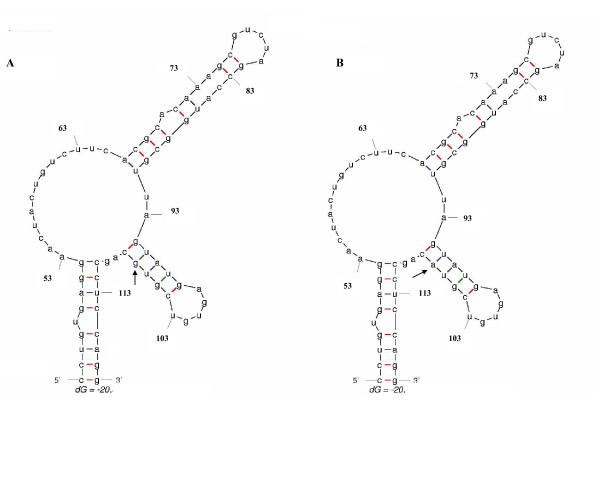
**Prediction of stem-loop II IRES RNA secondary structure**. *mfold *results of IRES stem-loop II are shown. Numbers in the figure denote nucleotide positions, Δ*G *obtained for the structures are shown on the bottom of the figure. In (A) *mfold *results for consensus type 1 strains isolated in South America is shown. (B) shows *mfold *results for signature consensus sequences.

**Figure 5:**
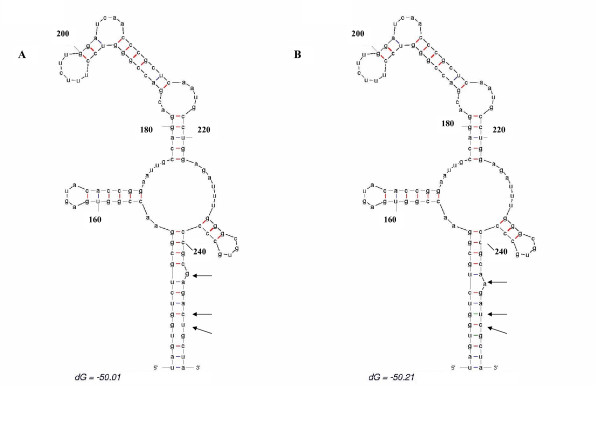
**Prediction of stem-loop III IRES RNA secondary structure**. *Mfold *results of IRES stem-loop III are shown. The rest same as Fig. 4.

To observe how these substitutions may affect IRES secondary RNA structure, predicted secondary structures of HCV IRES domains II and III of consensus dataset sequences of type 1 strains isolated in South America (background dataset) and consensus signature sequence dataset (query dataset) were compared. The results of these studies are shown in Figs. 4 and 5, respectively.

As it can be seen in Fig. 4, the predicted secondary structure of domains II of background and signature consensus sequences give similar structures. Nevertheless, mutation A107 in the sequence signature might help to stabilize a buckle in the structure by base pairing with U75 (compare Figs. 4A and 4B).

In the case of IRES stem-loop III predicted secondary structure, similar structures have also been obtained for background and signature sequences (see Fig. 5). Nevertheless, mutations in stem-loop III does not seem to have a particular effect in loop III folding (compare Figs. 5A and 5B).

## Discussion

Phylogenetic tree analysis of the 5'NCR from HCV strains isolated in South America revealed that genotype 1 is the most predominant in that region, in agreement with previous results [7]. There are no previous reports on the genetic variation of HCV circulating in Bolivia. All Bolivian strains enrolled in these studies have been clearly assigned to genotype 1. Although more studies will be needed in order to have a definitive picture on the degree of genetic heterogeneity of HCV strains circulating in Bolivia, the results of these studies suggests that genotype 1 might also be prevalent in that country (see Fig. 1A). In the case of Colombia, previous studies suggested the presence of genotype 1 and 3 [17]. This is in agreement with the results found in the present study. Interestingly, the phylogenetic analysis revealed the presence of genotype 4 in Colombia for the first time (see Fig. 1A, bottom). This genotype is prevalent in the Middle East [2] and not particularly in the South American region, although genotype 4 has been also found in Argentina [7]. More studies will be needed to address the epidemiological situation of this genotype in Colombia.

The phylogenetic analysis of HCV strains isolated in South America also revealed the presence of a new genetic lineage in HCV type 1 strains (Fig. 1A). These results are in agreement with previous ones obtained for type 1 HCV isolates circulating in Central and South America [8-12]. These previous data have suggested the presence of a distinct type 1 HCV sub-population in South America and a diversification of HCV in that region. In this study, we have analyzed more than 150 HCV strains isolated in South America. The results of this work revealed that the third type 1 sub-population observed in the phylogenetic tree analysis of the HCV strains isolated in South America is in fact due to the presence of a particular nucleotide signature sequence (Fig. 2 and Table 1). This sequence signature is frequent enough to be detected in a phylogenetic tree analysis as a distinct type 1 sub-population (see Fig. 1A). Nevertheless, when the same analysis is carried out in type 1 HCV strains isolated in Europe or North America, only two genetic lineages are observed which correspond to the major type 1 sub-types (see Fig. 1B and 1C).

Sequence signature pattern analysis has been useful for epidemiological linkage, to corroborate transmission link hypothesis or sequence relatedness studies [18-21]. The identification of a sequence signature in the 5'NCR of type 1 HCV strains isolated in South America may permit a more in-depth study on the molecular epidemiology of HCV in this region.

Nevertheless, more studies will be needed to determine the extent of distribution of this particular signature. BLAST studies, on the other hand, have shown that only type 1 HCV strains circulating in the South American region have 100% similarity to the nucleotide sequence signature found in that region.

HCV, as many other RNA viruses, replicates as complex mutant distributions termed quasispecies [22-25]. Quasispecies dynamics is characterized by continuous generation of variant viral genomes, competition among them, and selection of the fittest mutant distributions in any given environment [23]. The coexistence of distinct type 1 HCV subpopulations is consistent with quasispecies dynamics, and suggests that multiple coexisting subpopulations may occupy different regions on a fitness landscape to allow the virus to adapt rapidly to changes in the landscape topology. This, in turn, may allow the virus to adapt to its human host populations.

The 5'NCR, even though is one of the most conserved part of the virus genome, shows a quasispecies distribution with minor variants observed in the population [26] (Fig. 3). Since virus particles in serum are likely to be released from the liver but also from compartments such as lymphocytes or dendritic cells, it has been suggested that the sequence diversity found in the IRESs may reflect their translational activity and tropism for these compartments [27-29].

If all this is correct, the results of these studies may also be related to these facts. Owing to the error-prone nature of the HCV polymerase, mutations are expected to occur randomly distributed over the 5'NCR. However, only mutations compatible with replication and translation can be propagated. Whether the stem-loop II and III mutations observed confer a survival advantage or disadvantage *in vivo *remains unknown. Nevertheless, the *in silico *predicted RNA secondary structures of IRES stem-loops suggest that some mutations in the signature sequence might have an effect in IRES structure. Further work with HCV replicons containing the observed signature mutations may help to clarify this point.

The unique structure of the HCV IRES makes it an attractive target for the development of antiviral agents directed against this RNA element [30]. Mapping sequence signatures in that region may help to understand their effects in HCV IRES functions.

## Conclusion

Phylogenetic analysis revealed the presence of a sequence signature in the 5'NCR of type 1 HCV strains isolated in South America. This signature is frequent enough in type 1 HCV populations circulating South America to be detected in a phylogenetic tree analysis as a distinct type 1 sub-population. The coexistence of distinct type 1 HCV subpopulations is consistent with quasispecies dynamics, and suggests that multiple coexisting subpopulations may allow the virus to adapt to its human host populations.

## Methods

### Serum samples

Serum samples were obtained from 7 volunteer blood donors from Banco de Sangre de Referencia Departamental, La Paz, Bolivia, 14 volunteer blood donors from Banco de Sangre de la Cruz Roja, Bogotá, Colombia and 26 HCV chronic patients from Servicio Nacional de Sangre, Montevideo, Uruguay. All patients tested positive in an enzyme immunoassay from Abbott, used accordingly to manufacturer's instructions. All patients were from La Paz, Bogotá and Montevideo, respectively. For epidemiological data of Bolivian, Colombian and Uruguayan strains, see Table 2.

**Table 2:** Origins of Bolivian, Colombian and Uruguayan HCV strains

**Name**	**Accession Number**	**Patient ID**	**Age**^a^	**Sex**
Col2	[EMBL:AM269927]	451103850	39	Male
Col3	[EMBL:AM269928]	451209881	54	Male
Col4	[EMBL:AM269929]	451202563	20	Female
Col5	[EMBL:AM269926]	451200819	30	Female
Col11	[EMBL:AM269930]	451202594	49	Female
Col14	[EMBL:AM269931]	451201641	23	Female
Col18	[EMBL:AM269932]	451201714	24	Female
Col20	[EMBL:AM269936]	451204950	29	Male
Col24	[EMBL:AM269937]	451201157	28	Female
Col25	[EMBL:AM269925]	451201208	31	Female
Col26	[EMBL:AM269933]	451205577	25	Female
Col28	[EMBL:AM269934]	451209889	21	Female
Col29	[EMBL:AM269935]	451203054	25	Male
Bol1	[EMBL:AM400873]	13183	46	Male
Bol2	[EMBL:AM400874]	12713	48	Female
Bol3	[EMBL:AM400875]	12577	42	Male
Bol4	[EMBL:AM400876]	13410	42	Male
Bol5	[EMBL:AM400877]	12573	43	Male
Bol6	[EMBL:AM400878]	13177	42	Male
Bol7	[EMBL:AM400879]	13322	9	Male
Uru1	[AM709653]	H1	29	Male
Uru2	[AM709654]	H2	36	Male
Uru4	[AM709655]	H4	27	Male
Uru6	[AM709656]	H6	32	Male
Uru7	[AM709657]	H7	41	Male
Uru7A	[AM709676]	HCV7A	Adult	Male
Uru7B	[AM709671]	HCV7B	Adult	Male
Uru8	[AM709658]	H8	34	Male
UruG8	[AM709676]	HCVG8	Adult	Female
Uru9	[AM709659]	H9	78	Male
Uru14	[AM709660]	H14	39	Male
Uru17	[AM709661]	H17	28	Male
Uru18	[AM709662]	H18	29	Male
Uru20	[AM709663]	H20	20	Male
Uru23	[AM709664]	H23	25	Male
Uru26	[AM709665]	H26	56	Male
Uru27	[AM709666]	H27	59	Male
Uru29	[AM709667]	H29	57	Male
UruHCV20	[AM709668]	HCV20	68	Female
Uru41	[AM709669]	HCV21	32	Male
Uru51	[AM709673]	HCV51	55	Male
Uru60	[AM709675]	HCV60	Adult	Female
Uru64	[AM709672]	HCV64	Adult	Female
Uru66	[AM709678]	HCV66	29	Male
Uru72	[AM709670]	HCV72	Adult	Male
Uru99	[AM709474]	HCV99	Adult	Male

^a ^Adult means older than 18.

### PCR amplification of 5'NCR of HCV strains

The 5'NCR of the HCV genome from samples that were reactive in the enzyme immunoassay were amplified by PCR, as previously described [31,32]. To avoid false positive results, the recommendations of Kwok and Higuchi [33] were strictly adhered to. Amplicons were purified using QIAquick PCR Purification Kit from QIAGEN, according to instructions from the manufacturers.

### Sequencing of PCR amplicons

The same primers used for amplification were used for sequencing the PCR fragments, and the sequence reaction was carried out using the Big Dye DNA sequencing kit (Perkin-Elmer) on a 373 DNA sequencer apparatus (Perkin-Elmer). Both strands of the PCR product were sequenced in order to avoid discrepancies. 5'NCR sequences from position 62 through 285 (relative to the genome of strain AF009606, sub-type 1A) were obtained. For sequence accession numbers of Bolivian, Colombian and Uruguayan HCV strains, see Table 2.

### Phylogenetic tree analysis

5'NCR from HCV strains previously reported in South America, Europe and North America were obtained from the LANL HCV Database [14]. Sequences were aligned using the CLUSTAL W program [34]. Phylogenetic trees were generated by the neighbor-joining method under a matrix of genetic distances established under the Kimura-two parameter model [13], using the MEGA3 program [35]. The robustness of each node was assessed by bootstrap resampling (1,000 pseudo-replicas).

### Signature pattern analysis

Signature pattern analysis identifies particular sites in amino acid or nucleic acid alignments of variable sequences that are distinctly representative of a query set relative to a background set. We employed the method described by Korber & Myers [36] as implemented in the VESPA program [37]. Sequences in the query and background datasets where aligned using the CLUSTAL W program [34] and then transformed to the FASTA format using the MEGA 3 program [35]. The query set was formed by 19 type 1 HCV sequences isolated in South America and representative of the third genetic lineage identified in the phylogenetic tree analysis (see Fig. 1A). The background set was formed by 19 type 1 HCV sequences isolated in South America. The same studies were performed using background sets of 19 type 1 HCV strains isolated in Europe or North America. The threshold was set to 0 (the program will use the majority consensus sequence in the query dataset for calculations) or 0.5 (the program will require that the signature nucleotides be included at least in the 50% of the sequences in the query set to be included for calculations). Both thresholds gave the same results (not shown). For accession numbers of strains included in query and background datasets see Table 3.

**Table 3:** HCV strains included in query and background datasets for sequence signature studies^a^

**Dataset**^b^	**Strains included**
Query	[EMBL:AM266927], URU1, URU2, URU4, URU6, URU8, URU9, URU14, [EMBL:AM269928], [EMBL:AM269929], [EMBL:AM269930], [EMBL:AM269931], [EMBL:AM269932], [EMBL:AM269933], [EMBL:AM269934], [EMBL:AM269935], [EMBL:AM269936], [EMBL:DQ077818], [EMBL:DQ313454].
Background1	URUG7B, [EMBL:M84855], [EMBL:M84856], URU11, [EMBL:AB154179], [EMBL:AY576553], [EMBL:AY576557], [EMBL:DQ319979], [EMBL:M84838], [EMBL:M84839], [EMBL:M84841], [EMBL:AF077232], [EMBL:AF077236], [EMBL:AJ291457], [EMBL:AJ438617], [EMBL:AJ438619], [EMBL:AF011751], [EMBL:DQ010313], [EMBL:L34386].
Background2	[EMBL:AY576557], [EMBL:AY576576], [EMBL:DQ319979], [EMBL:DQ313980], [EMBL:DQ319983], [EMBL:M84838], [EMBL:M84840], [EMBL:M84841], [EMBL:M84842], [EMBL:Z84279], [EMBL:Z84280], [EMBL:D31722], [EMBL:AB154177], [EMBL:AB154178], [EMBL:Z84284], [EMBL:AB154179], [EMBL:AB154180], [EMBL:D31723], [EMBL:D31724].
Background3	[EMBL:AF009606], [EMBL:AY446036], [EMBL:AY446039], [EMBL:AY446043], [EMBL:AY446044], [EMBL:AY446049], [EMBL:AY446050], [EMBL:AY446051], [EMBL:AY446052], [EMBL:AY446053], [EMBL:AY446067], [EMBL:AY446068], [EMBL:DQ061296], [EMBL:DQ061297], [EMBL:DQ061299], [EMBL:L34377], [EMBL:L34385], [EMBL:L34388], [EMBL:L34389].

^a ^Strains previously reported are indicated by accession number, strains reported in this work are indicated by name.

^b ^Background datasets 1, 2 and 3, correspond to strains isolated in South America, Europe or North America, respectively.

### Sequence similarity studies

Sequence similarity among query signature strain URU2 and all HCV strains of all types, isolated elsewhere, was established using BLAST program [38], using the HCV LANL Database [14].

### Prediction of RNA secondary structure

Secondary structure prediction was done by the method of Zuker & Turner [39], as implemented in the *mfold *program (version 3.2) [40]. The core algorithm of this method predicts a minimum free energy, Δ*G*, as well as minimum free energies for foldings that must contain any particular base pair. The folding temperature was set to 37°C. Ionic conditions was set to 1M NaCl, non divalent ions. Base pairs that occur in all predicted folding structures are colored black. Otherwise, base pairs are assigned in a multi-color mode that displays precisely what foldings contain that base pair.

## Competing interests

The author(s) declare that they have no competing interests.

## Authors' contributions

JC and GM conceived and designed the study. MFG, KG, ARM, and AGS contributed with HCV samples from Colombia, Bolivia and Argentina, respectively, and to the discussion of the results found in the study. GM, MM and FL obtained PCR amplicons and sequences from Bolivian and Colombian strains. MM contributed to the discussion of the results found. RC, LL, RR, MPM and LG obtaining PCR amplicons and sequences from Uruguayan strains. JC wrote the paper. All authors have read and approved the final document.
